# Improving oral bioavailability of acalabrutinib using polymer–lipid hybrid nanoparticles: design, optimization, and *in vivo* pharmacokinetic evaluation

**DOI:** 10.1039/d5na00386e

**Published:** 2025-09-17

**Authors:** Swagata Sinha, Punna Rao Ravi, Sahadevan Rajesh Rashmi, Lakshmi Koumudi Devaraju

**Affiliations:** a Department of Pharmacy, Birla Institute of Technology and Science Pilani Hyderabad Campus, Jawahar Nagar, Kapra Mandal, Medchal District Telangana 500078 INDIA rpunnarao@hyderabad.bits-pilani.ac.in

## Abstract

Acalabrutinib (ACP) is one of the first-in-line treatments for hematological malignancies with minimal adverse drug reactions. However, ACP has low and variable bioavailability due to pH-dependent solubility, CYP3A4 metabolism, and P-gp efflux. This research aims to modify the dissolution behavior of ACP and improve its oral bioavailability through formulation of polymer–lipid hybrid nanoparticles (PLHNs). ACP-loaded PLHNs (ACP-PLHNs) were prepared by the emulsification–solvent evaporation method using a high shear homogenizer and optimized using a spherical and rotatable circumscribed central composite design. The optimized ACP-PLHNs exhibited a spherical morphology with an average particle size of 150.2 ± 10.7 nm, a PDI of 0.284 ± 0.06, and sufficient drug loading (20.79 ± 3.61%). *In vitro* dissolution studies showed that over 50% of ACP was released from the PLHNs at pH 1.2 within 4 h, reaching nearly 100% release by 24 h. While, at pH ≥ 4.5, 43–55% of ACP was released by 8 h, with sustained release observed for up to 2 days. *In vivo* hemolysis assay indicated that ACP-PLHNs were safe for oral administration. Storage stability studies over 6 months demonstrated optimal physico-chemical stability when stored at 5 °C. *In vivo* oral pharmacokinetic studies revealed that ACP-PLHN nanosuspension resulted in a 3.41-fold increase in bioavailability (*p* < 0.001) compared to the conventional ACP suspension, along with a >2-fold increase in drug distribution towards the spleen (*p* < 0.001), a critical target site for B-cell accumulation and proliferation.

## Abstract 2

Acalabrutinib loaded polymer lipid hybrid nanoparticles, developed and optimized using a DoE approach, were able to improve the oral bioavalability of the molecule. This highlights the potential of the PLHN technology to improve therapeutic performance of acalabrutinib.

## Graphical abstract

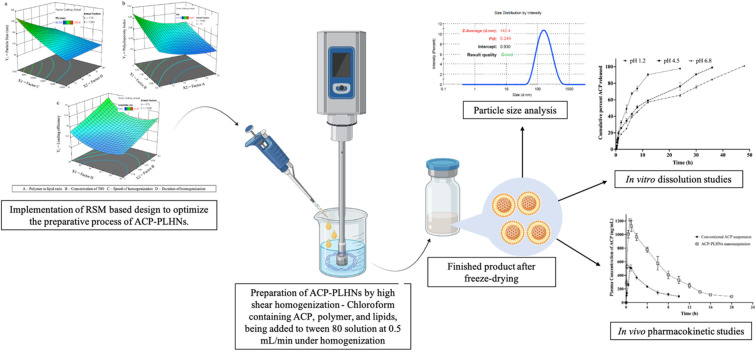

## 1 Introduction

In recent years, the development of advanced drug delivery systems has gained significant attention due to its potential to revolutionize the treatment of various diseases. Among these innovative approaches, polymer–lipid hybrid nanoparticles (PLHNs) have emerged as a promising platform for encapsulating and delivering hydrophobic molecules, which often face various biopharmaceutical challenges. PLHNs leverage the unique properties of both polymers and lipids with polymers playing a critical role in controlling drug release from the nanoparticles and imparting good physical stability to the nanoparticles, while the lipids enhance biocompatibility and cellular internalization of the nanoparticles.^1^ The formulation components can be natural or synthetic or semi-synthetic. Polylactic acid, polycaprolactone (PCL), chitosan, and poly(lactic-*co*-glycolic acid) (PLGA) are some of the commonly used polymers in the preparation of PLHNs. Among lipids, 1,2-dipalmitoylsn-glycero-3-phosphocholine (DPPC); 1,2-dilauroyl-*sn*-glycero-3-phosphocholine (DLPC); phosphatidyl choline from soy, sunflower or egg; and fatty acids such as steric, palmitic, myristic acid; *etc.* can be used alone or in combination to formulate PLHNs.

Acalabrutinib (ACP), available as Calquence (100 mg capsules/tablets) by AstraZeneca, Cambridge, UK is indicated for treating chronic lymphocytic leukemia (CLL) or small lymphocytic lymphoma.^2,3^ Despite its clinical superiority, ACP faces several biopharmaceutical challenges – pH dependent solubility which limits its presence at the site of absorption (mainly in the small intestine having a pH of 6.8) in a solubilized state, ultimately affecting absorption. Moreover, it is a substrate for P-gp efflux systems and is extensively metabolized by cytochrome P450 3A4 (CYP3A4) enzymes.^4,5^ These factors contribute to a low and variable oral bioavailability (25 ± 14%). Earlier studies have shown that PLHNs offer a promising strategy to overcome the solubility, bioavailability, and stability issues of molecules with such properties. Patel *et al.* (2023) developed ibrutinib-loaded PLHNs using PLGA, lipoid S 100 and DSPE-m-PEG 2000^6^ which achieved sustained drug release over 48 h, resulting in a 22% increase in relative bioavailability compared to a conventional aqueous suspension of ibrutinib. Similarly, the poor and variable bioavailability of raloxifene was addressed by formulating PLHNs with lecithin and chitosan, resulting in a 4.2-fold increase in bioavailability and enhanced endocytic uptake of the drug.^7^ PLHNs of docetaxel have addressed challenges related to high toxicity,^8^ poor pharmacokinetics,^9^ and pharmacodynamic activity.^10,11^

Most improvements in drug delivery systems stem from their physical characteristics, such as particle size, size distribution, drug-holding capacity, drug release under various physiological conditions, and colloidal stability (both at the site of administration and in systemic circulation). In this context, Design of Experiments (DoE) provides a more comprehensive framework for optimizing complex drug delivery systems compared to the traditional one-factor-at-a-time (OFAT) approach, accounting for the complex interplay of multiple factors and their interactions, enabling more precise and efficient optimization of carrier systems.^12–14^

Our research group has previously formulated and evaluated various nanocarriers for ACP, including nanocrystals,^15^ solid lipid nanoparticles (SLNs),^16^ and nanostructured lipid carriers (NLCs).^17^ While each of these systems provided certain benefits, PLHNs were adopted in this study due to their hybrid structural composition, combining the solubilizing capacity and biocompatibility of lipids with the controlled-release properties and stability of polymers. This study optimizes ACP-loaded PLHNs (ACP-PLHNs) using DoE and examines the impact of material and process parameters on critical quality attributes of ACP-PLHNs. Additionally, the article covers *in vitro* dissolution/release behaviour, *in vivo* oral pharmacokinetics, and spleen distribution studies. Overall, these findings highlight the potential of ACP-PLHNs as an effective delivery system, offering improved therapeutic outcomes.

## 2 Materials and animals

ACP was provided as a gift sample by MSN Laboratories Pvt. Ltd, Hyderabad, India. 1,2-Dipalmitoyl-*sn*-glycero-3-phosphocholine (16 : 0 PC, LECIVA-DPPC) and lecithin USP NF (LECIVA-S90) were sourced from VAV Life Sciences Pvt. Ltd, Mumbai, India. Polycaprolactone (PCL, 14 000 Da) was obtained from Sigma Aldrich Pvt. Ltd, Mumbai, India. Tween 80 (T80) and chloroform were purchased from TCI Pvt. Ltd, Hyderabad, India. Other reagents, chemicals and solvents were sourced from SRL Pvt. Ltd, Mumbai, India. Water, from an in-house Milli-Q water purification System (Merck Millipore, MA, USA), was used for all experiments.

Male Wistar rats (220–250 g) were purchased from Jeeva Life Sciences, Hyderabad, India (1757/PO/ReBiBt/S/14/CPCSEA). The experimental protocol (BITS-Hyd/IAEC/2021/15) was evaluated and approved by the Institutional Animal Ethics Committee (IAEC).

## 3 Methods

### 3.1. Preformulation and preliminary trials

Surfactants (poloxamer 188, poloxamer 407, T80, polyvinyl alcohol, and polyvinyl pyrrolidone K30) were screened and evaluated for their ACP solubilizing ability and formulation impact. Polymers and lipids (lecithin S90, DSPC, DSPG, and DPPC) were chosen for their functionality and ability to form PLHNs with optimal particle size (PS), polydispersity index (PDI), loading efficiency (%LE), and stability. Preliminary trials defined critical quality attributes (CQAs), critical material attributes (CMAs), and process parameters (CPPs). Based on the number of CMAs and CPPs, an appropriate experimental design was selected.

### 3.2. Experimental design using DoE

#### 3.2.1. Implementation of design

The critical factors (independent variables) – polymer to lipid ratio; concentration of surfactant; speed of homogenization; and duration of homogenization – affecting the CQAs (dependent variables) (PS; PDI; and %LE) were taken up directly for a response surface methodology (RSM) based design. A circumscribed central composite design (cCCD) with the level of axial points (*α*) at 2 was created using Design Expert software (ver. 13.0.5.0; Stat-Ease Inc., Minneapolis, USA) (Table S1). A set of 28 experimental runs (16 factorial runs, 8 axial point runs, and 4 center point runs) was suggested by the software. The best-fit regression equation for each quality attribute was assessed using ANOVA (at 5% level of significance, *α* = 0.05) to evaluate model significance and potential lack-of-fit. Additionally, statistics for goodness-of-fit and the impact of any transformation were evaluated using Box–Cox power transformation plots for each CQA and other diagnostic plots. The impact of the most significant factors on responses was assessed using 3D response surface plots.

#### 3.2.2. Desirability solution and validation of the model

A desirability function was used to optimize ACP-PLHNs, aiming for desirable PS, PDI, and %LE. This function converts multiple response variables/quality attributes into a composite score (0 to 1), enabling simultaneous handling of different responses with different objective functions (to maximize, minimize or achieve a target value for each CQA). The final optimized formulation was determined through experiments based on the suggested parameters. To validate the regression model, observed results from six independent runs were compared with predicted values and their confidence intervals. A Wilcoxon signed-rank test was conducted, at the 5% level of significance (*α* = 0.05), to statistically compare the observed responses of six independent formulations with their predicted values from the regression equation of each CQA. The regression models were deemed valid when there was no significant difference between observed and predicted values.

### 3.3 Preparation of ACP-PLHNs

ACP-PLHNs were formulated by the emulsification–solvent -evaporation method using a high shear homogenizer (Polytron PT3100D, Kinematica AG, Malters, Switzerland).^7,18^ The organic phase (OP) contained 20 mg of ACP, 20 mg of PCL, and 5 mg each of DPPC and lecithin dissolved in 1 mL of chloroform. The aqueous phase (AP) was prepared with 0.83% w/v T80 in Milli-Q water, maintaining a temperature of 55 °C. The OP was added at a rate of 0.5 mL min^−1^ into the AP under homogenization at 14 900 rpm while maintaining the temperature at 23–25 °C. The dispersion was subjected to homogenization for a total duration of 17 min. The resultant nano-dispersion was rested for 60 min at 24 ± 1 °C to allow evaporation of the residual amount of chloroform. Furthermore, the nano-dispersion was centrifuged (using a fixed angle rotor at 16 627 × *g*, 10 °C, 30 min), decanted and the resultant pellet was washed twice with 0.25% w/v T80 solution. Finally, the washed pellet was redispersed in MilliQ water containing 0.25% w/v T80 and 2% w/v mannitol (cryoprotectant) and subsequently freeze-dried in a freeze dryer (Coolsafe 110-4, LaboGene A/S, Allerød, Denmark).

### 3.4. HPLC-UV based methods of analysis

Samples from both *in vitro* studies (drug content, stability studies and drug release studies) and *in vivo* pharmacokinetics were analyzed using validated HPLC-UV methods using a HPLC system (Shimadzu Prominence, Shimadzu Corporation, Kyoto, Japan) with a PDA-UV detector (SPD-M20A). A Phenomenex Luna® Omega polar C-18 column (150 × 4.6 mm, 5 μm) served as the stationary phase and a combination of 10 mM ammonium acetate buffer (pH 3.5) and methanol served as the mobile phase. For *in vitro* samples, the column oven was maintained at 55 °C, with a mobile phase ratio of 48 : 52 (aqueous: organic), a flow rate of 1.05 mL min^−1^ and an injection volume of 50 μL. Detection was carried out at 250 nm. The method exhibited linearity from 0.05–5 μg mL^−1^, with limits of quantification (LOQ) and detection (LOD) of 4.32 and 1.46 ng mL^−1^, respectively.

To analyse the drug in the samples obtained from *in vivo* pharmacokinetic studies, a previously reported HPLC method was utilized.^15^ ACP was extracted from the plasma and spleen samples by protein precipitation using acidified methanol. The supernatant was concentrated under vacuum, and reconstituted in the mobile phase (60 : 40 buffer to methanol) before injecting into the HPLC system. The column temperature was maintained at 53 °C, with a mobile phase flow rate of 1.1 mL min^−1^ and an injection volume of 50 μL. For plasma, the method was linear from 0.08–5 μg mL^−1^, with LOD and LOQ values of 24.84 and 74.53 ng mL^−1^, respectively.

### 3.5. Characterization of optimized formulation

#### 3.5.1. Size and morphology analysis

The PS and PDI of freshly prepared and reconstituted ACP-PLHNs were measured by dynamic light scattering (DLS) using a zeta sizer (NANO-ZS, Malvern Pananalytical Ltd, Worcestershire, UK). Samples were diluted 150×, equilibrated for 120 s at 25 °C and analyzed using an He–Ne laser with the detector placed at a backscatter angle of 173°. The average of three measurements was recorded for each sample. The same instrument was used for measurement of zeta potential (ZP) using a Smoluchowski model.

Morphology of ACP-PLHNs was assessed using a field emission scanning electron microscope (FE-SEM, FEI Apreo LoVac, Oregon, USA). 20–50 μL of freshly prepared diluted suspension was cast as a thin film, dried, and sputter-coated with a gold–aluminum mixture (10 nm thickness) and viewed at various magnifications to capture the image of the ACP-PLHNs.

#### 3.5.2. Drug loading (%LE) and entrapment efficiency (%EE)

The %LE and %EE of ACP-PLHNs (*n* = 6 batches) were determined by indirect and direct methods (eqn (1) and (2)). Fresh suspensions were centrifuged (using a fixed angle rotor at 16 627 × *g*, 10 °C, 30 min). The supernatant (containing free ACP) was used for indirect measurement, and the pellet was dissolved in methanol for direct analysis. In both the methods, the samples were suitably diluted and analyzed by the HPLC-UV method (Section 3.4).1
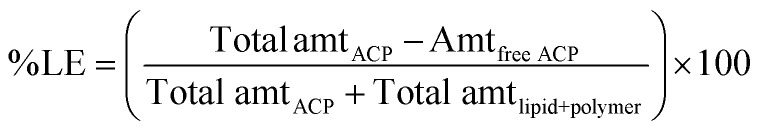
2
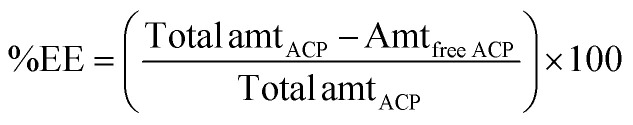
where Total amt_ACP_ = ACP added per batch of ACP-PHLNs; Amt_free ACP_ = amount of ACP present in the supernatant; Total amt_lipid+polymer_ = cumulative amount of DPPC, lecithin, and PCL added per batch of ACP-PHLNs. Since no significant difference was observed between the two estimations methods, the indirect method was chosen for subsequent calculations.

#### 3.5.3 Thermal analysis and powder X-ray diffraction (p-XRD) analysis

Pure ACP; a physical mixture of PCL, DPPC, and lecithin; pure mannitol; and freeze-dried ACP-PLHNs were subjected to thermal analysis using DSC-60 (TA-60 WS, Shimadzu, Kyoto, Japan). For pure ACP, an amount equivalent to ACP in freeze-dried PLHNs (0.8 mg ACP, ∼20.7% of 4 mg) was analyzed. After equilibration at 30 °C for 2 min in the sample chamber, the samples were examined in a nitrogen atmosphere between 30 and 300 °C (10 °C min^−1^ heating rate). The physical nature of entrapped ACP was further evaluated by p-XRD (ULTIMA IV Rigaku, MA, USA), measuring between 10 and 80° (3° min^−1^) with a copper X-ray source and scintillation counter detector.

#### 3.5.4. Presence of residual solvents

Residual chloroform was quantified using a gas chromatograph (GC-2010 plus, Shimadzu Corporation, Kyoto, Japan) with a flame ionization detector. The stationary phase was a Spinco Tech Enable EB-1 column (30 m × 0.25 mm), maintained at 40 °C for 1 min, then ramped to 120 °C (30 °C min^−1^), followed by a further ramp to 210 °C (30 °C min^−1^). Nitrogen and air were used as mobile phase (54.6 mL min^−1^ total flow and 1.01 mL min^−1^ column flow). A 1 : 50 split ratio was applied, with the injection port and detector temperatures set to 220 °C and 250 °C, respectively. A calibration curve for chloroform was constructed in the range of 1.5 to 120 ppm using DMSO as the solvent. To detect residual chloroform, freeze-dried ACP-PLHNs were dissolved in DMSO (3 mg mL^−1^) and analyzed.

#### 3.5.5 *In vitro* dissolution studies

The dissolution and release of both bulk ACP and freeze-dried ACP-PLHNs (containing ∼100 mg ACP) were evaluated using a USP Type II dissolution apparatus. Dissolution studies were conducted under three different conditions: 250 mL of 0.1 N HCl buffer (pH 1.2) mimicking fasted stomach pH, 250 mL of acetate buffer (pH 4.5) mimicking fed stomach pH, and 900 mL of phosphate buffer solution (PBS, pH 6.8) representing small intestinal pH. In addition, to mimic the blood/plasma pH, dissolution studies were also conducted in 500 mL of PBS (pH 7.2). To maintain sink conditions, 0.5% w/v sodium dodecyl sulfate (SDS) was added to dissolution medium at pH ≥ 4.5. In all studies, the temperature was maintained at 37 ± 2 °C with stirring at 75 rpm. Samples were collected at specific time-intervals, replaced with fresh media, centrifuged (using a fixed angle rotor at 15 373 × *g*, 5 min) and analyzed using the HPLC-UV method (Section 3.4). The dissolution data were fitted to zero-order, first-order, Higuchi square root kinetics, and Korsmeyer–Peppas models to determine drug release kinetics and mechanism. The best-fit model was selected based on highest regression coefficient (*r*^2^).

#### 3.5.6 Effect on RBC morphology

Before *in vivo* studies, the safety of ACP-PLHNs was evaluated by examining their effect on red blood cell (RBC) morphology. Reconstituted freeze-dried ACP-PLHNs (30 mg kg^−1^) were orally administered to healthy rats (*n* = 3). Blood samples were collected at 0.5, 0.75, and 1 h. RBCs were isolated and re-suspended in cold PBS to form 2% v/v suspension. Triton X (1% v/v) was used to lyse RBCs (positive control), and 0.9% saline served as the negative control. RBCs, including controls, were fixed with 2% v/v glutaraldehyde for 1 h, 4 °C in the dark. After washing with cold PBS, RBCs were dehydrated using ethanol solutions (30%, 50%, 80%, 90%, and 100% v/v) followed by rehydration. The final suspension was cast as a thin film, dried, sputter-coated and analyzed using FE-SEM.

#### 3.5.7 Storage stability analysis

The influence of storage conditions on key features of freeze-dried ACP-PLHNs was evaluated according to ICH Q1A(R2) guidelines. Products were stored under three conditions: 45 ± 2 °C/75 ± 5% RH, 25 ± 2 °C/60 ± 5% RH, and 5 ± 2 °C, for 6 months. During storage, PS, PDI, and %EE were measured at regular intervals. The results are presented as mean ± SD for PS and PDI, and as %deviation relative to the fresh formulation (0 days) for %EE. The *in vitro* release behavior of the most stable formulation was compared with that of the freshly prepared formulation at pH 7.2 following the method detailed in Section 3.5.5 and evaluated using the similarity factor (eqn (3)).3
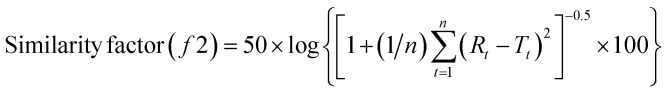
where *n* = number of time points considered in the dissolution study; *R*_*t*_ = dissolution values of ACP-PLHNs at time ‘*t*’ for the freshly prepared formulation (before storage), and *T*_*t*_ = dissolution values of ACP-PLHNs at time ‘t’ after 6 months of storage.

#### 3.5.8 *In vivo* pharmacokinetic (PK) and tissue distribution studies

Prior to the *in vivo* studies, the rats were grouped and transferred to clean cages for a 10–12 h fasting period, during which only water was provided to prevent drug–food interactions and coprophagia.

##### 3.5.8.1 Single dose oral PK studies

Oral PK studies with ACP-PLHN nanosuspension, reconstituted with Milli-Q water, were conducted at 30 mg kg^−1^ (dosing volume of 4 mL kg^−1^, *n* = 3 animals). To calculate absolute and relative bioavailability, PK data for i.v. bolus administration of simple ACP solution and oral administration of conventional ACP suspension were obtained from previously reported data by the same research group^16^ (Fig. S2, SI file). 300 μL of blood was collected at pre-determined time points followed by sample processing and analysis using the method described in Section 3.4. PK parameters were determined using Phoenix WinNonlin software (version 8.4.0.6172; Pharsight Corporation, NC, USA).

##### 3.5.8.2 Drug distribution studies to the spleen

To quantify ACP in the spleen, *n* = 4 rats were orally administered ACP-PLHN nanosuspension at the same dose as used in oral PK studies. Spleen tissues (*n* = 2 rats were sacrificed at each time point) were collected at two time points: *T*_max_ of conventional ACP suspension (0.75 h) and at *T*_max_ + twice the half-life of ACP in rats (3.75 h). Spleen tissues were cleaned, weighed, minced; and homogenized separately at 8000 rpm using a T10 basic ULTRA-TURRAX® (IKA India Pvt. Ltd, Mumbai, India). The samples were processed and analyzed as described in Section 3.4.

#### 3.5.9 Statistical analyses

Statistical analysis was performed to evaluate the significance of observed experimental data. Student′s *t*-test was used to compare the data obtained from studies which involved two groups. Data obtained from the DoE, including model fitting and evaluation of interaction effects, were analyzed *via* analysis of variance (ANOVA) using Design-Expert software (version 13). The agreement between predicted and observed values for each CQA was evaluated using the Wilcoxon signed-rank test. All statistical tests were evaluated at a 5% level of significance (*α* = 0.05).

## 4 Results and discussion

### 4.1 Preparation of ACP-PLHNs

#### 4.1.1 The preliminary trials

##### 4.1.1.1 Selection of formulation components

The literature suggests that PLHNs consist of a polymer matrix for drug entrapment, a lipid component for biocompatibility, and a surfactant layer for stability.^19^ Solubility analysis (Table S2) showed that T80 offered the least solubility to ACP enabling effective entrapment of the drug in the nanoparticles and reducing the drug loss into the aqueous phase during the manufacturing by emulsification. PCL, a hydrophobic, semi-crystalline polymer, offers good solubility and biocompatibility with minimum side effects.^20,21^ Depending on the molecular weight of PCL used in the preparation of nanoparticles, it also offers customizable degradation rates and improved mechanical properties to the formulation.^21^ PCL also exhibits higher drug encapsulation capacity compared to other widely used polymers like polyglycolide, polylactide, or their copolymer PLGA.^22^ During the preliminary trials, ACP-PLHNs prepared with DSPC, DSPG, and DPPC showed PSs of 311.3 nm, 168.2 nm, and 157.2 nm, respectively, with PDIs of 0.50, 0.36, and 0.41, and %LE of 4.32%, 7.2%, and 6.84%, respectively. Of all the formulations prepared in the preliminary trials, the ACP-PLHNs formulated with DPPC remained stable at room temperature, with a PS of 153.7 nm and a PDI of 0.367 after 24 h. To further improve the formulation properties, cholesterol and lecithin were combined with DPPC, where lecithin provided better %LE and offered good physical stability to the nanoparticles. Thus, the final formulation included PCL as the polymer, DPPC and lecithin as lipids, chloroform as the solvent, and T80 as the surfactant.

##### 4.1.1.2 Determination of CQAs and critical factors

A review of previous studies on implementing DoE in formulating PLHNs identified PS, PDI, ZP, %LE, and %EE as potential CQAs.^23–25^ For the current study, the goal was to improve the PK performance of ACP administered *via* ACP-PLHNs. For a nanoparticulate drug delivery system administered through the oral route, average PS affects gastro-intestinal uptake and target site delivery,^26^ while PDI indicates aggregation behavior, influencing release rates, stability, and targeting.^27,28^ ZP influences particle aggregation and interaction with biological tissues and cellular membranes. However, for ACP-PLHNs, neither the lipids nor the surfactant used for formulating the nanoparticles exhibited a characteristic charge. Therefore, the ACP-PLHNs exhibited a near neutral ZP. Although %LE and %EE are determined similarly (eqn (1) and (2)), they differ in their significance. %LE measures the quantity of drug incorporated into the carrier system and depends on the drug to carrier ratio, affinity of the drug for the carrier systems and the manufacturing method.^29^ For this study, the selected CQAs were – PS (*Y*_1_); PDI (*Y*_2_); and %LE (*Y*_3_).

A one-step manufacturing method using high shear homogenization was selected for the preparation of ACP-PLHNs, where emulsification, size reduction, and solvent evaporation occurred simultaneously. Preliminary trials to determine the critical factors and their levels affecting the CQAs are listed in Table S2. The factors selected for optimization were – polymer to lipid ratio (A), concentration of T80 (B), speed of homogenization (C) and duration of homogenization (D).

##### 4.1.1.3 Experimental design using DoE

From the preliminary trials, four critical factors were identified as affecting the three CQAs. Given the complexity and cost of running experiments with these factors, a separate screening phase was deemed unnecessary. Therefore, the factors were directly incorporated into a RSM-based optimization design, a cCCD with an *α* value of 2 to ensure sphericity and rotatability. A total of 28 experimental runs were conducted in a single block (Table 1). PS (*Y*_1_) followed a reduced two-factor interaction model with a natural logarithmic transformation, while both PDI (*Y*_2_) and %LE (*Y*_3_) followed reduced quadratic models with inverse square root and square root transformations, respectively. The model *F*-values were 12.26 for *Y*_1_, 7.16 for *Y*_2_, and 6.44 for *Y*_3_, indicating that the models were significant. The probability of obtaining such large *F*-values due to noise was 0.01%, 0.02%, and 0.06% for *Y*_1_, *Y*_2_, and *Y*_3_, respectively. The significance of the model and the factors affecting the responses were further evaluated using ANOVA (Table 2).

**Table 1:** The cCCD experimental runs along with the values obtained against each experimental run^a^

Standard run number	Factor A	Factor B	Factor C	Factor D	*Y*_1_ =PS	*Y*_2_ = PDI	*Y*_3_ = %LE
3	1	2	7500	10	175.6	0.56	56.20
23	1.75	1.35	11 300	5	193.2	0.41	33.16
17	0.25	1.35	11 300	15	84.5	0.67	19.96
16	2.5	2	15 000	20	83.59	0.51	39.05
18	3.25	1.35	11 300	15	148.1	0.29	12.49
5	1	0.7	15 000	10	143.9	0.28	3.83
24	1.75	1.35	11 300	25	118.1	0.32	47.31
27	1.75	1.35	11 300	15	153.8	0.36	10.13
1	1	0.7	7500	10	313.1	0.40	37.56
26	1.75	1.35	11 300	15	140.1	0.25	9.55
8	2.5	2	15 000	10	98.24	0.32	12.82
21	1.75	1.35	3800	15	250.4	0.45	52.42
2	2.5	0.7	7500	10	353.2	0.51	47.21
11	1	2	7500	20	122.0	0.40	31.78
28	1.75	1.35	11 300	15	144.6	0.27	6.49
25	1.75	1.35	11 300	15	135.3	0.23	3.26
13	1	0.7	15 000	20	143.9	0.28	61.83
12	2.5	2	7500	20	161.8	0.28	31.30
10	2.5	0.7	7500	20	191.9	0.33	21.54
22	1.75	1.35	18 800	15	133.9	0.42	45.52
20	1.75	2.65	11 300	15	164.6	0.63	0.64
15	1	2	15 000	20	121.0	0.63	14.63
19	1.75	0.05	11 300	15	168.8	0.25	50.01
4	2.5	2	7500	10	355.6	0.45	15.94
14	2.5	0.7	15 000	20	116.0	0.22	51.01
6	2.5	0.7	15 000	10	147.0	0.25	34.60
7	1	2	15 000	10	93.9	0.44	13.36
9	1	0.7	7500	20	189.6	0.38	37.85

a Standard run number defines a standard label to describe the geometric location of the run in the space. Factor A = polymer to lipid ratio; B = concentration of T80 (%w/v); C = speed of homogenization (rpm); and D = duration of homogenization (min). Temperature of AP was maintained at 55 °C; ratio of OP to AP was maintained at 1 : 25; and the rate of addition of OP to AP was maintained at 0.5 mL min^−1^, across all the trials. The responses are reported as the mean of 3 independent measurements.

**Table 2:** ANOVA table including the model terms for the CQAs as obtained from the regression equations^a^

Source	Probability values (*p*-values) obtained from ANOVA of the regression equations		
	*Y*_1_ =PS	*Y*_2_ = PDI	*Y*_3_ = %LE
Model	<0.0001	0.0002	0.0006
A	0.0924	0.0051	—
B	0.0165	0.0003	0.0073
C	<0.0001	0.0956	0.2528
D	0.0023	0.2429	0.1478
AC	0.0548	—	—
BC	—	0.007	—
CD	0.0153	0.029	0.0278
A^2^	—	0.057	—
C^2^	—	0.0372	0.0011
D^2^	—	—	0.0052
Lack of fit	0.217	0.691	0.108

a *p*-values <0.05 indicate that the model terms are significant. Factor A = polymer to lipid ratio; B = concentration of T80 (%w/v); C = speed of homogenization (rpm); and D = duration of homogenization (min).

##### 4.1.1.4 Effect on PS

**Fig. 1:**
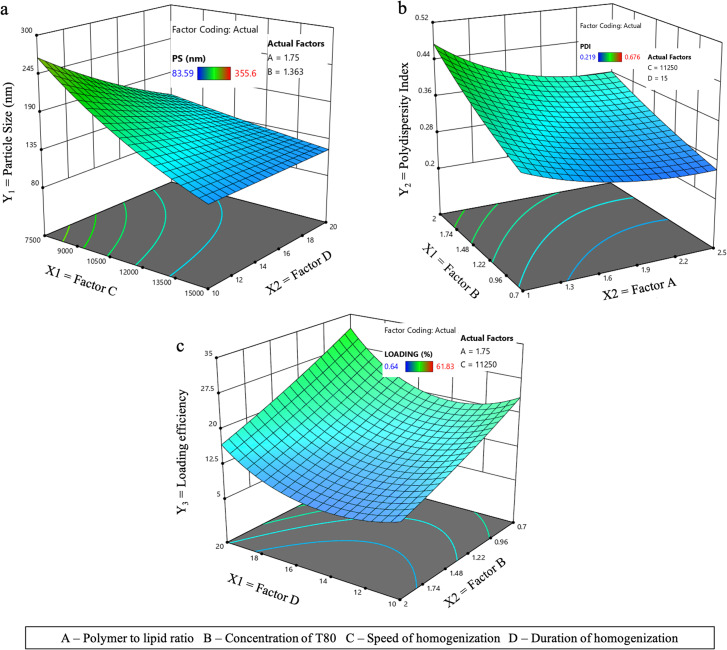
The response surface 3D plot depicting the effect of the most significant factors on PS (a); PDI (b); and %LE (c).

PS of ACP-PLHNs was most significantly influenced by the speed and duration of homogenization. At a fixed polymer to lipid ratio (1.75) and T80 concentration (1.363% w/v), a 2-fold increase in homogenization speed reduced PS by 4-fold (Fig. 1a), with minimal impact from the duration of homogenization. This effect was due to the higher energy density at increased speeds, which reduced emulsion droplet size by enhancing shear stress.^30^ Furthermore, at this high energy state, chloroform readily diffused through AP and evaporated, eventually turning the nanodroplets into nanoparticles. A similar effect was observed at lower speed (∼7500 rpm) where the energy to reduce the emulsion droplet size was provided by longer homogenization duration. The accuracy of the transformation function for PS was confirmed by Box–Cox plot (Fig. S1a).

4 log_10_(PS) = 2.19 + 0.0314*A* − 0.0464*B* − 0.1135*C* − 0.0617*D* − 0.0443*AC* + 0.0575*CD*.

The analysis of factor coefficients in eqn (4) revealed a consistent trend with Fig. 1a. From eqn (4), it can be inferred that the polymer fraction had a positive effect of the PS while, a negative impact on the PS.

##### 4.1.1.5 Effect on PDI

Fig. 1b illustrates the influence of two key factors – concentration of T80 and polymer to lipid ratio – on PDI, while keeping homogenization speed and duration constant at 11 250 rpm and 15 min, respectively. Generally, an increase in the concentration of surfactant lowers interfacial tension, reduces the likelihood of agglomeration of globules, and encourages a unimodal distribution of globules (and thereby the particles formed) in an emulsion. However, for ACP-PLHNs, an increase in the concentration of T80 led to an increase in the PDI (Fig. 1b and eqn (5)).

This was likely due to the uneven energy distribution during homogenization at higher surfactant concentration (which can alter the viscosity of the AP), causing some particles to shrink to smaller sizes while others remained larger.5
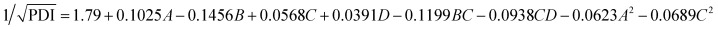


Notably, eqn (5) shows that an increase in homogenization speed alone reduces PDI. Furthermore, the equation also suggests that PDI is inversely affected by the polymer to lipid ratio and homogenization duration.

The Box–Cox plot (Fig. S1b) confirms the accuracy of the transformation function for PDI.

##### 4.1.1.6 Effect on %LE

The %LE of ACP-PLHNs was significantly affected by the concentration of T80 and the duration of homogenization (Fig. 1c). An increase in the concentration of T80 led to a decrease in ACP loading, as higher surfactant availability in the AP caused more drug to diffuse into the AP. Fig. 1c shows a complex effect of homogenization duration on %LE. When homogenization was maintained at 11 250 rpm for 10 min, larger globules were formed, allowing greater ACP loading. However, as homogenization duration increased from 10 to 13 min, the globules broke due to high energy, destabilizing the system and causing ACP to leak into the AP. Beyond 13 min, the energy was sufficient for chloroform evaporation, promoting faster particle formation and an immediate integration of ACP into the polymer–lipid matrix, improving %LE.6
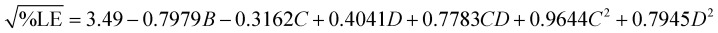


Eqn (6) indicates that homogenization speed exerts a negatively influence on %LE, as higher speeds can disrupt the ACP-PLHN structure and cause subsequent drug distribution drug into the AP. The suitability of model transformation was confirmed by the Box–Cox plot given in Fig. S1c.

##### 4.1.1.7 Desirability runs and validation of the optimized condition

The target parameters set for ACP-PLHNs were – PS < 250 nm, PDI < 0.4, and %LE > 20%. No boundaries were set for the factors except homogenization speed, which was set between 10 000 and 15 000 rpm. The optimal solution provided by the software suggested preparing the formulation with a polymer-to-lipid ratio of 2 : 1 (20 mg PCL, 5 mg each of DPPC and lecithin), a T80 concentration of 0.83% w/v, a homogenized speed of 14 900 rpm and a homogenization duration of 17 min. Six independent formulations prepared under these optimized conditions revealed no significant differences between experimental and predicted values for the responses (based on the Wilcoxon signed rank test, the *p*-values for all the CQAs were >0.05. For PS, *p* = 0.326; for PDI, *p* = 0.517; and for %LE, *p* = 0.337), with the mean observed values of each response falling within their respective predicted confidence intervals. The RSD values for each response in the six independent formulations were <5%, demonstrating high reproducibility, validating the predictability of the regression equations of the response variables.

### 4.2 Physical and morphological evaluation

**Fig. 2:**
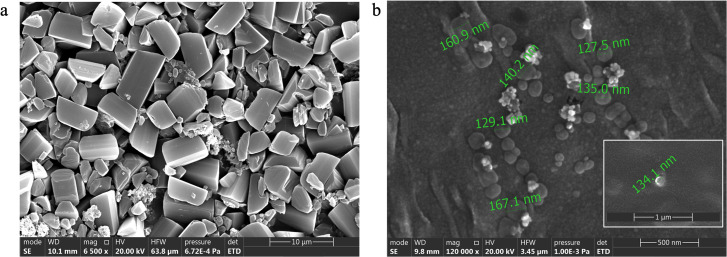
SEM images of bulk ACP (a) and ACP-PLHNs (b).

Freshly prepared ACP-PLHNs exhibited a PS of 136.9 ± 9.8 nm with a PDI of 0.26 ± 0.02 while, reconstituted freeze-dried ACP-PLHNs measured 150.2 ± 10.7 nm with a PDI of 0.28 ± 0.06. The ZP of ACP-PLHNs ranged from (−)5.48 to 12.6 mV. Fig. 2 illustrates that bulk ACP particles were trapezoidal in shape while, ACP-PLHNs were spherical.

### 4.3 Drug loading and entrapment efficiency

As discussed earlier, determining %LE and %EE is essential for estimating both the total amount of formulation to be administered and the amount of drug associated with these particles.^31^ The %LE and %EE values of the optimized ACP-PLHNs were found to be 20.79 ± 3.61% and 58.36 ± 9.10%, respectively.

### 4.4 Thermal and p-XRD analysis

**Fig. 3:**
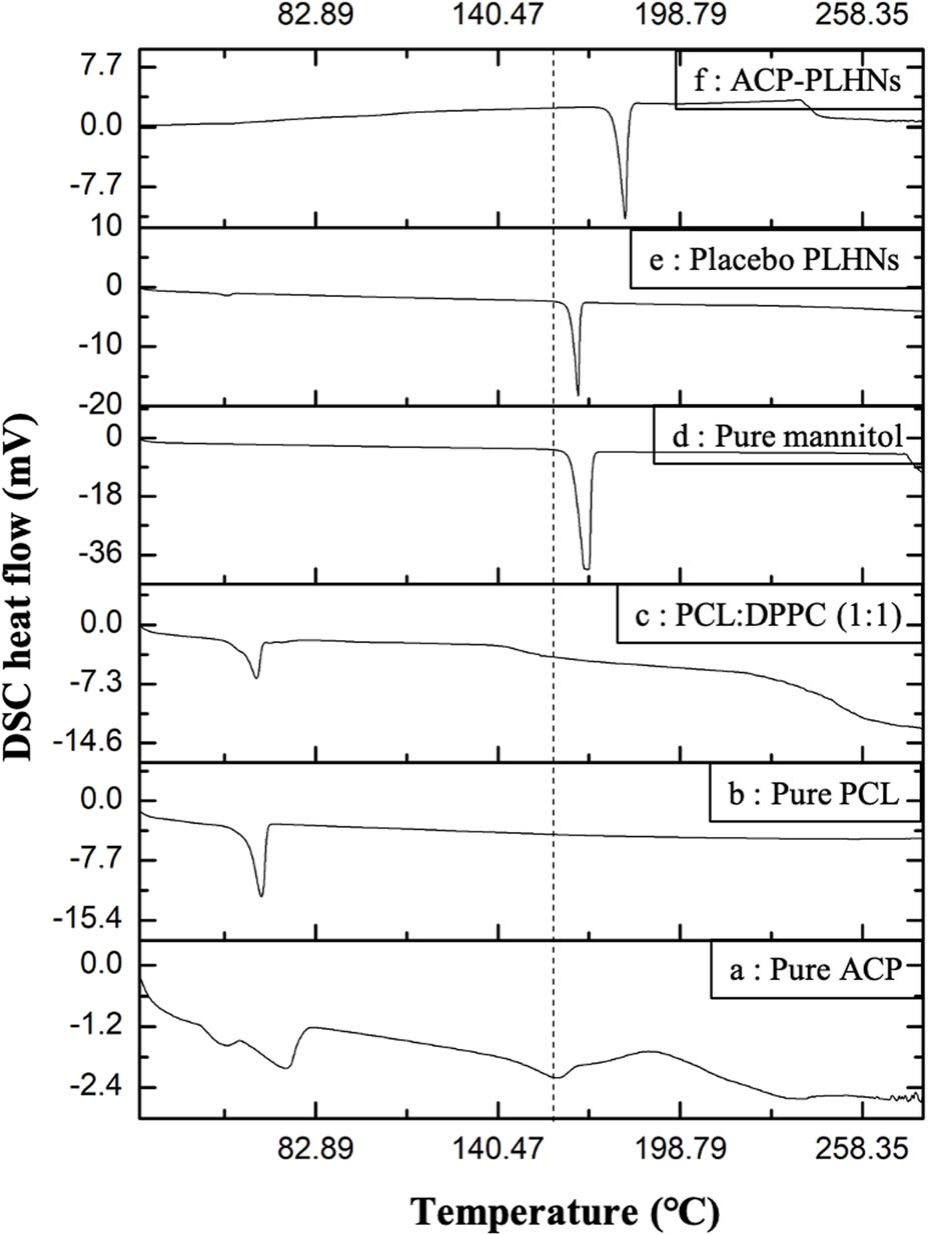
Overlay of thermograms from DSC analysis of pure ACP (a); pure PCL (b); a physical mixture of PCL and DPPC (c); pure mannitol (cryoprotectant) (d); freeze-dried placebo PLHNs (e); and freeze-dried ACP-PLHNs (f).

**Fig. 4:**
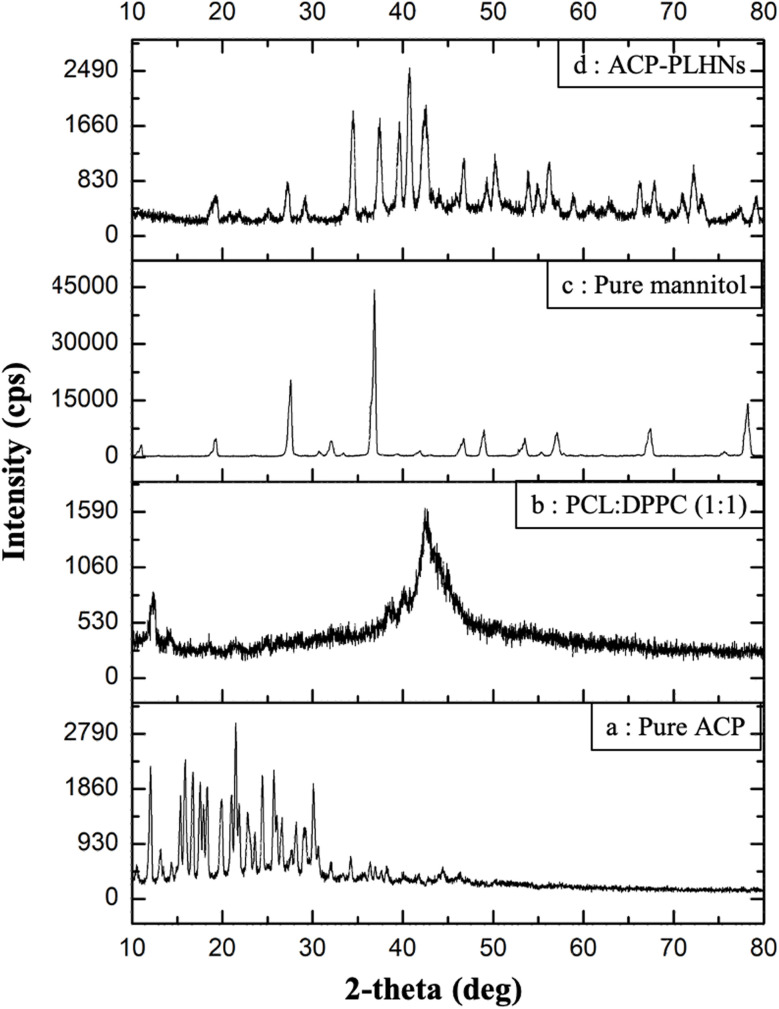
Overlay of diffraction patterns obtained from p-XRD of pure ACP (a); a physical mixture of PCL and DPPC (b); pure mannitol (cryoprotectant) (c); and freeze-dried ACP-PLHNs (d).

The thermogram of pure ACP showed peaks at 65 °C, 162 °C, and 198 °C, (Fig. 3a) corresponding to its “S” polymorph.^32^ Pure PCL (Fig. 3b) and the PCL–DPPC physical mixture (Fig. 3c) showed an endothermic peak near 60 °C. Pure mannitol exhibited an endothermic peak at 170 °C (Fig. 3d). The freeze-dried placebo PLHNs and ACP-PLHNs (Fig. 3e and f) did not exhibit characteristic DSC peaks as that of pure ACP (at 65 °C, 162 °C, and 198 °C), supporting incorporation of ACP within the nanoparticles at a molecular level. In p-XRD analysis, pure ACP exhibited characteristic peaks at 2-theta values of 10.5°, 12.1°, 15.8°, 24.4°, 25.7°, and 30.1° (Fig. 4a). The polymer–lipid physical mixture exhibited a broad peak around 43° (Fig. 4b). Pure mannitol displayed intense peaks at 27.5° and 36.3° (Fig. 4c), along with less intense peaks at 19°, 32°, and 45–80°. From the diffractogram of freeze-dried ACP-PLHNs (Fig. 4d) it could be inferred that ACP was either present in an amorphous state or a molecular form suggesting it's transformation in the nanoparticles, and further supporting the findings from DSC analysis.

### 4.5 Presence of residual solvents

**Fig. 5:**
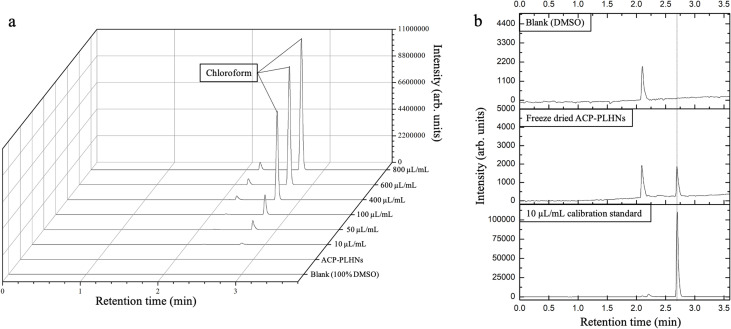
Overlay of the chromatograms between calibration standards, test sample (solubilized freeze-dried ACP-PLHNs), and blank DMSO (a). Comparative analysis between the lowest calibration standard (10 μL mL^−1^), test sample, and blank DMSO (b).

As per ICH Q3C(R8) guidelines, chloroform belongs to class II solvents with a permissible daily exposure (PDE) of 60 ppm per day^33^ which is also supported by the Agency for Toxic Substances and Disease Registry.^34^ The chloroform content in freeze-dried ACP-PLHNs was found to be <1.5 ppm (Fig. 5), which is significantly below the PDE limit, confirming that its levels in ACP-PLHNs are within the safety limits.

### 4.6 *In vitro* dissolution studies

**Fig. 6:**
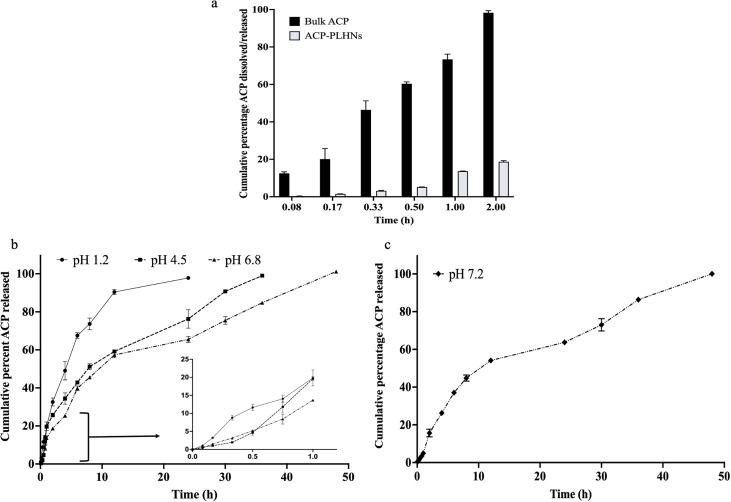
A comparative *in vitro* dissolution or release profile between bulk ACP and ACP-PLHNs in pH 6.8 buffer under sink conditions (a). Comparative dissolution profile of ACP-PLHNs under conditions representing different segments of GIT (b); and blood/plasma (c).

Pepin *et al.*, 2019 demonstrated that solubility of ACP sharply declines from 29.2 mg mL^−1^ at pH 1 to 0.159 mg mL^−1^ at pH 4.5 to 0.0523 mg mL^−1^ at pH 6.8.^35^ Hence, SDS was added to buffers with pH ≥ 4.5. The solubility of ACP in pH 6.8 buffer with 0.5% SDS was found to be 0.77 mg mL^−1^. Therefore, 0.5% SDS was sufficient to maintain sink conditions in all dissolution media with pH ≥ 4.5 in the volume in which the dissolution studies were conducted. Bulk ACP was completely dissolved (100% dissolution) within 2 h at pH 6.8 while only 20% drug release was observed in the case of ACP-PLHNs (Fig. 6a). Fig. 6b and c clearly indicate that PLHNs released ACP in a sustained manner for up to 48 h at pH 6.8 and 7.2. An initial release of 13–20% ACP under both the pH conditions (pH 6.8 and 7.2) within the first 2 h is likely due to the immediate release of surface-bound ACP on the nanoparticles. At pH 1.2, >50% ACP was released within 4 h, with complete release by 24 h. While, at pH ≥ 4.5, approximately 45% ACP was released by 8 h. The presence of DPPC and lecithin, can further enhance solubilization of ACP due to their emulsifying properties. The release profiles of these PLHNs were likely influenced by drug solubility, drug–polymer interactions, lipid and polymer degradation/erosion mechanisms, and the PS of nanoparticles.^36^

Furthermore, analysis of the dissolution data of ACP-PLHNs in different dissolution media using the Korsemeyer–Peppas model equation yielded a release exponent of 0.811 ± 0.05. This suggests that drug release followed non-Fickian transport, where the drug release is influenced by both drug diffusion from the nanoparticle matrix (of polymer + lipid) and by the polymer relaxation/swelling.^37^ The gradual polymer chain relaxation, along with the drug diffusion process, results in a time-dependent anomalous behavior. This release pattern suggests the possibility of a monolithic (matrix) system for ACP-PLHNs.

### 4.7 Effect on RBC morphology

**Fig. 7:**
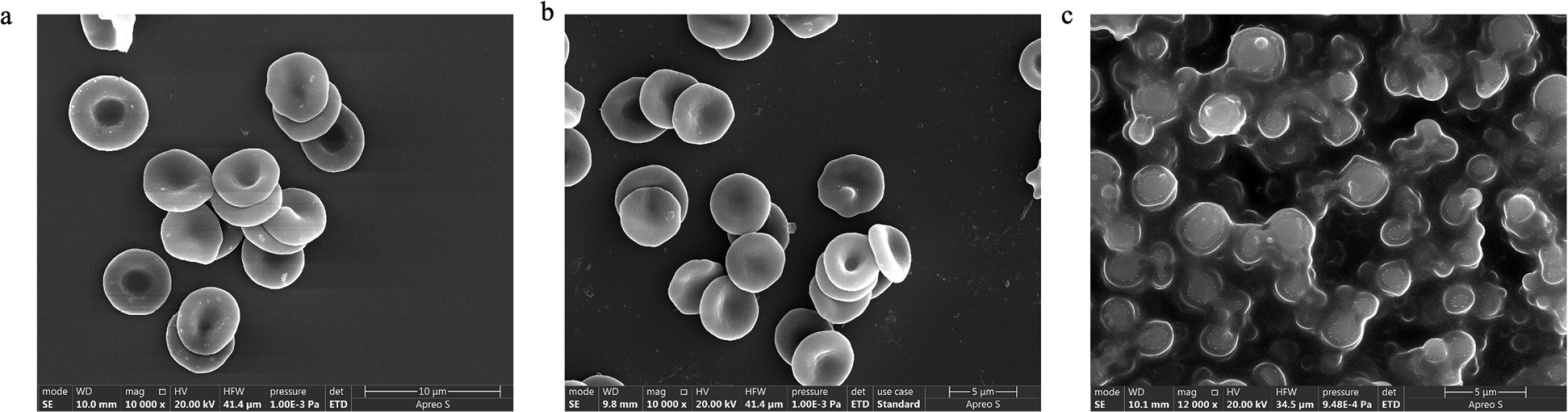
Morphology of RBCs collected from male Wistar rats after oral administration of ACP-PLHN nanosuspension (30 mg kg^−1^) (a) compared against the morphology of RBCs treated with the negative control (healthy RBCs) and (b) and the positive control (showing complete lysis) (c).

PLHNs can potentially enter systemic circulation by direct uptake through the enterocytes of the GIT, resulting in direct contact with RBCs. The interaction between ACP-PLHNs and RBCs can indicate their biocompatibility. Any morphological changes in RBCs can signal adverse interactions with ACP-PLHNs or its components inducing inflammatory responses or cell membrane damages. However, FE-SEM analysis of RBCs after oral administration of ACP-PLHNs revealed normal morphology (Fig. 7a depicts the morphology at 0.75 h), when compared to the negative control (Fig. 7b) and no traces of lysis like the positive control (Fig. 7c).

### 4.8 Storage stability

**Fig. 8:**
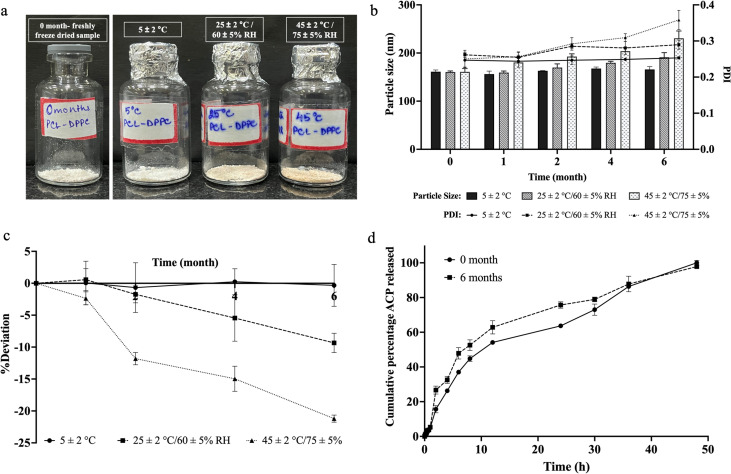
Stability analysis of freeze-dried ACP-PLHNs over 6 months, illustrating physical appearance (a), changes in the PS and PDI (b), and variations in %EE (c) under different storage conditions. A comparison between the dissolution profiles of the freshly prepared sample and the most stable sample after 6 months at pH 7.2 (d).

Storage stability analysis was conducted using freeze-dried ACP-PLHNs. After 6 months of storage, the formulation remained stable only at 5 ± 2 °C. Samples stored at higher temperature and/or humidity showed changes in powder color likely due to the oxidation of lipidic components (DPPC, lecithin), with the most significant changes at 45 ± 2 °C/75 ± 5% RH (Fig. 8a). No significant changes in PS (*p* > 0.05) were observed for samples stored at 5 ± 2 °C, while samples stored at higher temperature and humidity showed an increase in PS (>1.2 folds, (*p* < 0.05)) and PDI (0.301 to 0.376) (Fig. 8b). The %EE at 5 ± 2 °C remained relatively stable with a deviation of −3.97% to 3.81% over the 6-month study period. However, the samples stored under elevated environmental conditions showed a significant (*p* < 0.05) decrease in %EE. Particularly, the samples stored at 45 ± 2 °C/75 ± 5% RH showed a decrease in %EE after just one month of storage (Fig. 8c). Although PCL is a stable polymer, it begins to degrade around 37 °C which can be accelerated under elevated conditions due to molecular rearrangements.^38,39^ Additionally, the lipids can undergo phase transitions, shifting between gel and fluid states, which may result in the expulsion of ACP and reduction in its entrapped fraction. Based on these results, the refrigerated samples (5 ± 2 °C) were selected for dissolution testing at pH 7.2. A *f*2 value of 73.93% confirms the similarity between the drug release properties of the freshly prepared formulation and the formulation stored for 6 months at 5 ± 2 °C (Fig. 8d). This suggests that refrigeration effectively preserves the physicochemical characteristics of ACP-PLHNs.

### 4.9 *In vivo* PK and spleen distribution studies

**Fig. 9:**
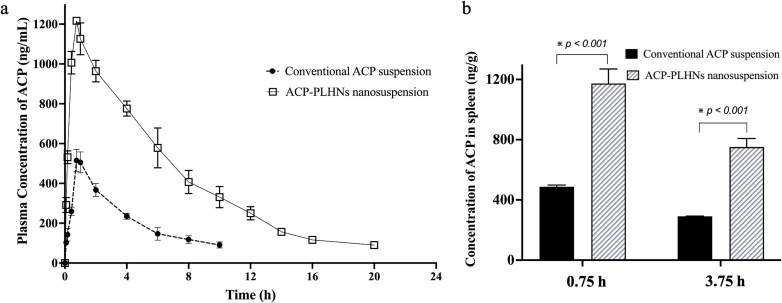
Comparative analysis of the *in vivo* PK profile obtained from oral administration of conventional ACP suspension and ACP-PLHNs nanosuspension to male Wistar rats (*n* = 3) (a) and comparison of ACP distribution towards the spleen after oral administration of conventional suspension and ACP-PLHN nanosuspension (*n* = 2 at each time point) (b).

The plasma concentration of ACP was significantly higher following the oral administration of ACP-PLHN nanosuspension compared to conventional ACP suspension (Fig. 9a). *C*_max_ and AUC_0−tlast_ increased by 2.22 and 3.36 times (*p* < 0.001), respectively. Furthermore, the absolute oral bioavailability of ACP-PLHN nanosuspension was also 3.41 times higher (*p* < 0.001) than that of conventional suspension. The increase in oral absorption of ACP-PLHNs could be due to several factors, including, the presence of DPPC and lecithin in ACP-PLHNs influencing the solubility of drug in the luminal fluids of the GIT; direct uptake of ACP-PLHNs into the systemic circulation due to their nanometric size circumventing the dissolution process and minimizing the chances of metabolism or efflux by CYP3A4 and P-gp enzyme systems due to the entrapment of ACP in the nanoparticles. Besides elevated ACP plasma-levels, ACP-PLHNs prolonged the residence time of the drug as reflected by MRT_0−tlast_ (5.90 ± 0.21 h) and reduced clearance values (Table 3) compared to conventional ACP suspension. This could be due to the slow degradation of the matrix system of the nanoparticles.

**Table 3:** PK parameters obtained after non-compartmental analysis of plasma concentrations following the oral administration of conventional ACP suspension and ACP-PLHN nanosuspension^a^

Parameters (units)		Conventional ACP suspension^^^	ACP-PLHNs nanosuspension
*T*_max_	h	0.75	0.8
*C*_max_	ng mL^−1^	558.25 ± 22.45	1223.31 ± 15.06*
*C*_last_	ng mL^−1^	94.53 ± 10.60	91.94 ± 8.63*
AUC_0−tlast_	h*ng mL^−1^	2447.85 ± 269.42	8233.96 ± 527.40*
*F*_abs_^#^	—	26.83 ± 1.35	83.83 ± 5.36*
*F*_rel_^##^	—	—	3.41 ± 0.56*
Cl/*F*_obs_	mL h^−1^	2434.96 ± 205.90	785.40 ± 75.39*
MRT_0−last_	h	3.64 ± 0.15	5.90 ± 0.21*

a All values are reported as the mean ± SD (*n* = 3), except for *T*_max,_ which is expressed as the median of 3 independent determinations. ^^^Data of conventional ACP suspension is reproduced from Sinha et. Al, 2024.^15^^#^Absolute bioavailability of both conventional ACP suspension and ACP-PLHNs nanosuspension were determined using *F*_abs_ = [((AUC_0−tlast_)_Oral_/(AUC_0−tlast_)_IV_) × (Dose_Oral_/Dose_IV_)] × 100. ^##^Relative bioavailability of ACP-PLHNs was determined using *F*_rel_ = (AUC_0−tlast_)_ACP−PLHNs nanosuspension_/(AUC_0−tlast_)_Conventional ACP suspension_ × 100. *PK parameters reported at *p* < 0.05.

The spleen is a crucial site for the accumulation and proliferation of CLL cells playing a key role in the pathophysiology of the disease.^40,41^ Oral administration of ACP-PLHN nanosuspension showed >2-fold higher (*p* < 0.001) ACP concentrations in the spleen compared to conventional suspension (values referred from a previously reported study by the same research group^16^ at both time points (Fig. 9b) which can potentially improve treatment outcomes as splenomegaly is a common symptom in patients suffering from CLL.^42^ The enhanced spleen accumulation observed is attributed to passive targeting, likely due to the nanoparticle size (∼136.9 ± 9.8 nm) and surface characteristics (neutral-to-slightly-negative surface charge), which favor uptake by the mononuclear phagocyte system (MPS). The spleen, being rich in phagocytic cells, naturally sequesters nanoparticles of this size range, which explains the increased accumulation.

## 5 Conclusion

In this study, ACP-PLHNs were successfully developed and optimized using a DoE approach to enhance the oral bioavailability of acalabrutinib. The optimized formulation exhibited favorable physicochemical characteristics, including a particle size of 130–160 nm, a PDI of less than 0.34, and a drug loading of 20.79 ± 3.61%. *In vitro* release studies demonstrated sustained drug release for over 48 h under both intestinal and systemic pH conditions. Stability studies confirmed that the formulation remained stable for at least six months under refrigerated conditions, likely due to the incorporation of PCL, which offers an advantage over previously developed pure lipid-based ACP-loaded nanoparticulate systems such as solid lipid nanoparticles and nanostructured lipid carriers.^16,17^*In vivo* pharmacokinetic analysis revealed a 3.41-fold increase in oral bioavailability and more than a 2-fold enhancement in spleen accumulation compared to conventional ACP suspension. Additionally, encapsulation of ACP within the PLHNs likely reduced its interaction with P-gp efflux transporters and intestinal metabolizing enzymes – an advantage over our previously formulated ACP nanocrystals.^15^ These findings highlight the potential of PLHN technology to significantly improve the therapeutic performance of ACP, particularly for spleen-targeted malignancies such as CLL and small lymphocytic lymphoma (SLL).

While the study demonstrates promising results, mechanistic investigations such as intestinal permeability or cellular uptake studies were not conducted and remain an important future direction. Overall, this work highlights the applicability of PLHNs as a viable platform for improving the clinical utility of poorly bioavailable anticancer agents like ACP.

## Author contributions

Conceptualization, formal analysis – Punna Rao Ravi (PRR) and Swagata Sinha (SS). Investigation, methodology, data curation – SS, PRR, Sahadevan Rajesh Rashmi (SRR), and Lakshmi Koumudi Devaraju (LKD). Writing – original draft preparation – SS. Writing – review and editing – SS and PRR. Supervision, project administration, resources, and funding acquisition – PRR.

## Conflicts of interest

The authors declare that they have no known competing financial interests or personal relationships that could have appeared to influence the work reported in this paper.

## Supplementary Material

NA-OLF-D5NA00386E-s001

## Data Availability

The datasets generated during and/or analysed during the current study are available in the manuscript. Additionally, the data supporting this article have been included as part of the SI. See DOI: https://doi.org/10.1039/d5na00386e.
