# Supplementary material for: Improving oral bioavailability of acalabrutinib using polymer–lipid hybrid nanoparticles: design, optimization, and in vivo pharmacokinetic evaluation
Source: Nanoscale Adv. 2025 Sep 17;7(21):6874–87. doi: 10.1039/d5na00386e (PMC12442105; doi:10.1039/d5na00386e)
Supplement: NA-007-D5NA00386E-s001 [file NA-007-D5NA00386E-s001.pdf]

Supplementary Information (SI) for Nanoscale Advances.
This journal is © The Royal Society of Chemistry 2025

Improving Oral Bioavailability of Acalabrutinib using Polymer Lipid Hybrid Nanoparticles : Design, Optimization, and in vivo Pharmacokinetic Evaluation.

Swagata Sinha[a], Punna Rao Ravi[a], Sahadevan Rajesh Rashmi[a], Lakshmi Koumudi Devaraju[a]*

[a]Department of Pharmacy, Birla Institute of Technology and Science Pilani, Hyderabad Campus, Jawahar Nagar, Kapra Mandal, Medchal District, Telangana, INDIA 500078.

Supplementary Data

- ## Implementation of design

The critical factors – polymer to lipid ratio (A); concentration of surfactant (B); speed of homogenization (C); and duration of homogenization (D) affecting the CQAs – PS (Y_1); PDI (Y_2); and %LE (Y_3) were taken up for a circumscribed central composite design (cCCD). The levels of the factors can be described as given in Table S1.

Table S1 : Critical factors, levels, and quality attributes used in the cCCD to optimize preparation of ACP-PLHNs.

Factor Code	Factors & units	High Level (+1)	Low Level (-1)	Alpha high (+α)	Alpha low (-α)	Center point level
A	Polymer to lipid ratio	2.5	1	3.25	0.25	1.75
B	Concentration of T80 (% w/v)	2.0	0.7	2.65	0.05	1.35
C	Speed of Homogenization (rpm)	15000	7500	18800	3800	11300
D	Duration of Homogenization (min)	20	10	25	5	15
**The CQAs of ACP-PLHNs**						
Y_1	PS (nm)	< 250 nm is desirable				
Y_2	PDI	< 0.6 is desirable				
Y_3	%LE	> 20% is desirable				

Table S2 : Solubility of ACP in aqueous solutions of different surfactants.

Surfactant	Concentration (% w/v)	ACP solubility (µg/mL)
Poloxamer 188	2	0.0202
	0.15	0.0075
Poloxamer 407	2	1.7391
	0.15	0.2095

Tween 80	2	0.1941
	0.15	0.0523
Polyvinyl alcohol	2	0.1087
	0.15	0.0137
Polyvinyl pyrrolidone	2	2.2728
	0.15	0.7479

- ## Determination of CQAs and critical factors

A number of preliminary trials (Table S3) were conducted to determine the critical factors and their levels affecting the CQAs of ACP-PLHNs.

Table S3 : Preliminary trials to determine the critical factors, their levels affecting the CQAs.

**Batch No.**	**Method attributes**		**High shear homogenizer parameters**	**CQAs**		
	**Polymer to lipid ratio**	**Concentration of T80 (% w/v)**		**PS (nm)**	**PDI**	**%LE**
01-PB	1:1	1	Speed = 10000 rpm<br>Time = 20 min	182.5	0.35	8.68
02-PB	2:1			208.1	0.41	10.42
03-PB	3:2			238.2	0.36	9.68
04-PB	2:1	0.5	Speed = 10000 rpm<br>Time = 20 min	189.1	0.37	10.26
05-PB		0.75		194.7	0.39	10.47
06-PB		1.5		247.1	0.48	9.37
07-PB		2		287.1	0.61	8.20
08-PB	2:1	1	Speed = 7500 rpm<br>Time = 20 min	198.2	0.50	9.91
09-PB			Speed = 12000 rpm<br>Time = 20 min	219.4	0.42	10.77
10-PB			Speed = 15000 rpm<br>Time = 20 min	179.4	0.41	8.93
11-PB	2:1	1	Speed = 10000 rpm<br>Time = 10 min	149.9	0.38	7.02
12-PB			Speed = 10000 rpm<br>Time = 15 min	130.9	0.35	8.23
13-PB			Speed = 10000 rpm<br>Time = 20 min	180.7	0.45	8.94

Note: CQAs = critical quality attributes; PS = particle size; PDI = polydispersity index; %LE = loading efficiency. Polymer = Polycaprolactone; Lipid = DPPC and lecithin. Temperature of AP was maintained at 55 °C and the rate of addition of OP to AP was maintained at 0.5 mL/min.

- ## Experimental design using DoE

The accuracy of transformation of PS (Y_1); PDI (Y_2); and %LE (Y_3) could be depicted by their respective Box-Cox power transformation plots (Fig. S1a, S1b, and S1c, respectively).

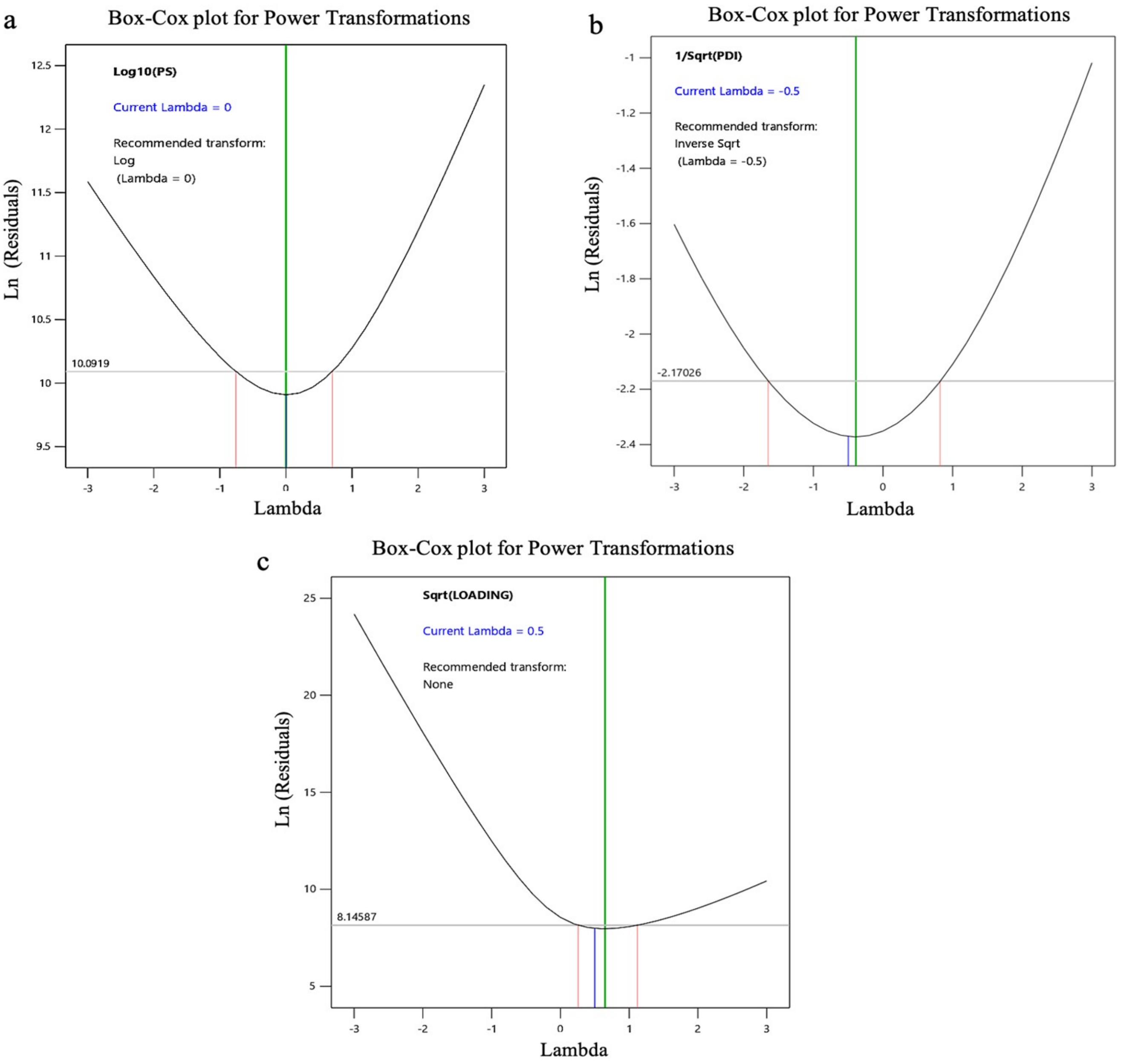

Fig. S1 : The Box-Cox power transformation plots depicting the goodness-of-fit of the selected model for the CRVs – PS (a); PDI (b); and %LE (c).

- **Pharmacokinetic profiles of the intravenous ACP solution and conventional ACP suspension** (1)

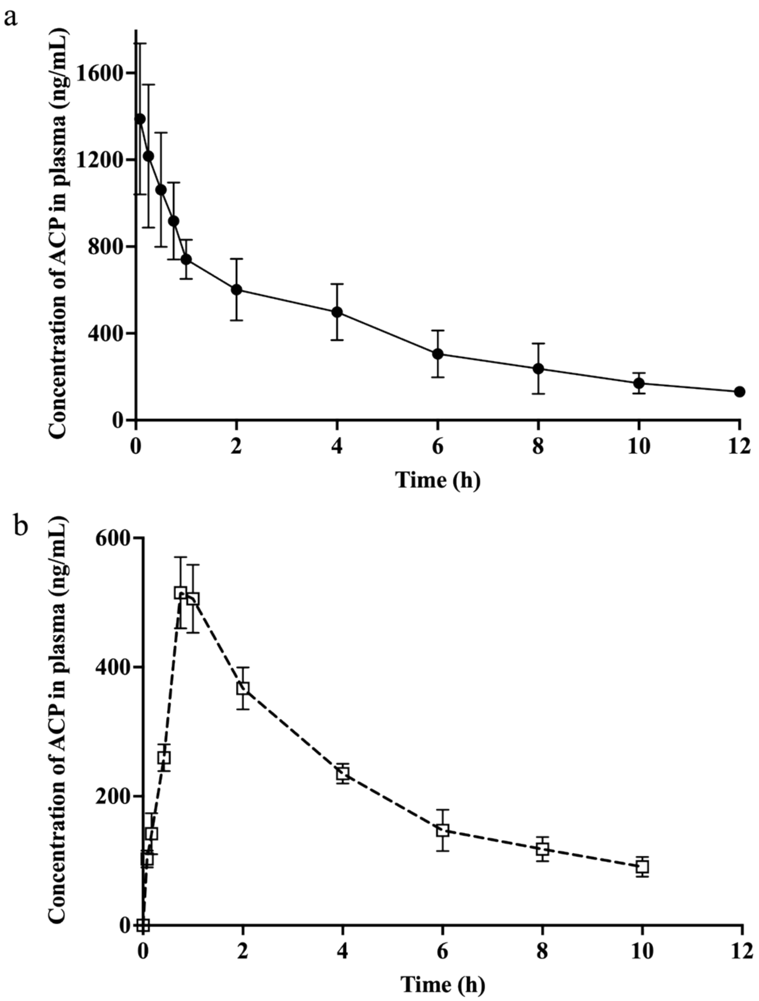

Fig. S2 : *In vivo* PK profile obtained from intravenous administration of ACP solution at the dose of 12 mg/Kg (a) and oral administration of conventional ACP suspension and ACP-PLHNs nanosuspension at the dose of 30 mg/Kg (b) to male wistar rats (n = 3).

Reference:

1. Sinha S, Ravi PR, Somvanshi M, SR R. Development and validation of a simple HPLC-UV-based bioanalytical method for estimation of acalabrutinib in rat plasma and its application in evaluation of drug loaded nanocrystal formulation. Sep Sci Plus. 2024;
